# TOP2A deficit-induced abnormal decidualization leads to recurrent implantation failure via the NF-κB signaling pathway

**DOI:** 10.1186/s12958-022-01013-1

**Published:** 2022-09-22

**Authors:** Huijia Fu, Wang Tan, Zhi Chen, Zi Ye, Yuhan Duan, Jiayu Huang, Hongbo Qi, Xiru Liu

**Affiliations:** 1 Department of Reproductive Medicine Center, The First Affiliated Hospital of Chongqing Medical University, No. 1 Youyi Road, Yuzhong District, Chongqing, 400016 China; 2 Chongqing Key Laboratory of Translational Medicine in Major Metabolic Diseases, Chongqing Medical University, Chongqing, 400016 China; 3 Joint International Research Laboratory of Reproduction and Development of Chinese Ministry of Education, Chongqing Medical University, Chongqing, 400016 China; 4 Department of Gynecology, Chongqing Traditional Chinese Medicine Hospital, Chongqing, 400021 No China; 5 Chongqing Key Lab of Ophthalmology, Chongqing Eye Institute, The First Affiliated Hospital of Chongqing Medical University, Chongqing, 400016 China; 6 Chongqing Key Laboratory of Maternal and Fetal Medicine, Chongqing Medical University, Chongqing, 400016 China; 7 Department of Obstetrics, Women and Children’s Hospital of Chongqing Medical University (Chongqing Health Center for Women and Children), 400010 Chongqing, China

**Keywords:** Recurrent implantation failure, Endometrial receptivity, Decidualization, DNA topoisomerase IIα, NF-κB

## Abstract

**Background:**

Successful implantation is a complex process that is influenced by embryo quality, endometrial receptivity, immune factors, and the specific type of in vitro fertilization protocol used. DNA topoisomerase IIα (TOP2A) is a well-known protein involved in cell proliferation; however, its expression and effect on the endometrium in recurrent implantation failure (RIF) have not been fully elucidated.

**Methods:**

The human endometrial tissues of healthy controls and patients with RIF were collected. A proteomic analysis was performed to evaluate the differentially expressed proteins between the RIF group and the fertile control group. The expression patterns of TOP2A in the human preimplantation endometrium of the patients with RIF were determined by immunohistochemical staining, Western blotting and qRT-PCR. TOP2A knockdown (sh-TOP2A) T-HESCs were generated using lentiviruses. The expression of TOP2A in T-HESCs was manipulated to investigate its role in decidualization. The TOP2A-related changes in decidualization were screened by mRNA sequencing in decidualized TOP2A knockdown and control T-HESCs and then confirmed by Western blotting and immunofluorescence staining. TOP2A-deficient mice were generated by injection of TOP2A-interfering adenovirus on GD2.5 and GD3.5.

**Results:**

We performed a proteomic analysis of endometrial tissues to investigate the potential pathogenesis of RIF by comparing the patients with RIF and the matched controls and found that TOP2A might be a key protein in RIF. TOP2A is ubiquitously expressed in both stromal and glandular epithelial cells of the endometrium. The data indicate that TOP2A expression is significantly lower in the mid-secretory endometrium of women with RIF. TOP2A expression was downregulated under stimulation by 8-bromo-cAMP and MPA. Ablation of TOP2A resulted in upregulated expression of decidual biomarkers and morphological changes in the cells. Mechanistic analysis revealed that TOP2A regulates the NF-κB signaling pathway in decidualized T-HESCs. The TOP2A-deficient mice exhibited lower fetal weights.

**Conclusions:**

Our findings revealed that abnormal expression of TOP2A affects decidualization and changes the “window of implantation”, leading to RIF. TOP2A participates in the processes of decidualization and embryo implantation, functioning at least in part through the NF-κB pathway. Regulating the expression of TOP2A in the endometrium may become a new strategy for the prevention and treatment of RIF.

## Introduction

In recent years, infertility has become an important medical and social problem worldwide. An increasing number of infertile patients require assisted reproductive technology (ART) to achieve a successful pregnancy. Studies show that 15% of couples of reproductive age worldwide suffer from infertility [1]. However, among Chinese couples of childbearing age, the prevalence of infertility is 25% [2], causing a series of public problems, including psychological distress, social stigma, economic pressure, and marital discord. The success of ART is closely related to each step in the process. Despite the rapid development of ART technology in recent years, approximately 10% of women seeking in vitro fertilization (IVF) treatment will experience recurrent implantation failure (RIF) [3]. RIF refers only to patients undergoing ART and describes a lack of successful implantation after repeated transfers of morphologically good-quality embryos into a normal uterus. However, there is no internationally accepted consensus definition of RIF [4]. Coughlan et al. suggested that RIF be defined as the failure to achieve a clinical pregnancy after the transfer of at least 4 good-quality embryos in a minimum of three fresh or frozen cycles in a woman under the age of 40 years [3]. Successful implantation is a complex process involving the following main factors: embryo quality, endometrial receptivity, immune factors, and the specific type of IVF protocol; among these, endometrial receptivity is a key element. Endometrial receptivity provides the embryo with the opportunity to attach, invade, and develop [5]. Endometrial receptivity is regulated by many factors, such as leukemia inhibitory factor [6], EHD1 [7], HOXA10 [8], and SOCS1 [9]. Although a series of advances have been made in this field, it is still impossible to accurately evaluate endometrial receptivity by monitoring specific indicators to improve the pregnancy rate. Therefore, it is necessary to identify key factors that affect endometrial receptivity and lead to recurrent implantation failure and to clarify their role and possible mechanism in the pathogenesis of RIF.

In our previous study [10] (Fig. S1), the GSE111974 dataset was downloaded from the Gene Expression Omnibus (GEO) at the National Center for Biotechnology Information. Specific R packages were applied to this model to screen out the differentially expressed genes (DEGs) in the patients with RIF compared with fertile controls. We identified 170 differentially expressed genes, of which 127 genes had upregulated expression and 43 had downregulated expression. Using the STRING database and Cytoscape to analyze the DEGs, we found that TOP2A (FC = 0.357, *p* = 0.0065) was the hub gene. TOP2A is an isoform of topoisomerase II (TOP2), which is essential for cellular viability and plays a key role in chromosome segregation [11]. TOP2A is a nuclear protein required for DNA replication and cell division [12]. This molecule is highly expressed during mitosis [13] and is a biomarker of cell proliferation [14]. Dong Y and colleagues [15] observed that hepatocellular carcinoma cell growth, migration, and invasion were suppressed after TOP2A knockdown. As a target of chemotherapeutic drugs, TOP2A has been proven to be widely involved in the aggressiveness and prognosis of a variety of human cancers [16]. Despite the well-known association between TOP2A and different diseases, the underlying role of TOP2A in RIF remains unclear. In the present study, we performed a proteomic analysis to investigate the potential pathogenesis of RIF and identified TOP2A as a key protein. We aimed to investigate the expression levels of TOP2A in the preimplantation endometrium of women with RIF. We also examined whether decreased TOP2A expression increases the secretion of prolactin (PRL) and insulin-like growth factor-binding protein 1 (IGFBP-1) and influences cytoskeletal formation during decidualization. We aimed to explore the role and mechanism of TOP2A in regulating endometrial receptivity for successful pregnancy.

## Materials and methods

### Cohorts

The present study was reviewed and preapproved by the Ethics Committee of Chongqing Medical University (Approval No. 2021–94). An informed consent form was signed by all participants at the beginning of the study. All procedures were performed in accordance with the Declaration of Helsinki.

Cohorts were recruited in the Reproductive Medicine Center of the First Affiliated Hospital of Chongqing Medical University between September 2018 and December 2019. Twelve female patients with RIF, along with twelve age- and BMI-matched female controls whose infertility was due to tubal infertility or male factors and achieved a clinical pregnancy after the first embryo transfer, were enrolled for subsequent analysis. All patients were 25–39 years of age and exhibited a regular menstrual cycle of 21–35 days. The diagnosis of RIF was based on the criteria reported by Coughlan C and colleagues in 2014 [3]. Individuals with polycystic ovarian syndrome (PCOS), hydrosalpinx, endometriosis, adenomyosis, intrauterine pathology (congenital uterine anomaly, fibroid, polyps, intrauterine adhesions), coupled with abnormal chromosomal complements, abortion tissues with abnormal karyotypes, or positivity for lupus anticoagulant or anticardiolipin antibodies were excluded. All endometrial biopsies were timed 7 days after the preovulatory LH surge.

In addition, we enrolled 24 females with infertility for routine hysteroscopy using the same exclusion and inclusion criteria described above. The subjects were divided into two groups (*n* = 12 per group): proliferative phase (cycle Days 5–13) and mid-secretory phase (cycle Days 20–23).

Prior to sample collection, none of the patients enrolled in this study had received hormonal therapy for at least 3 months. The demographic data (e.g., age and BMI) and clinical manifestations of all patients and controls were recorded (Table 1). Samples were obtained using an endometrial suction curette (Yajie, Jiangxi, China).

### Proteomic analysis

For iTRAQ analysis, fresh human endometrial tissues were collected, immediately frozen in liquid nitrogen, and stored in liquid nitrogen until use. Samples were sonicated three times on ice using a high-intensity ultrasonic processor (Scientz, Ningbo, China) in lysis buffer (8 M urea, 1% Protease Inhibitor Cocktail). The remaining debris was removed by centrifugation at 12,000 g at 4 °C for 10 min. Finally, the supernatant was collected, and the protein concentration was determined with a BCA Protein Assay Kit (Abcam, Shanghai, China) according to the manufacturer’s instructions. For digestion, the protein solution was reduced with 5 mM dithiothreitol for 30 min at 56 °C and alkylated with 11 mM iodoacetamide for 15 min at room temperature in darkness. The protein sample was then diluted by adding 100 mM TEAB to urea concentrations less than 2 M. Finally, trypsin was added at a 1:50 trypsin-to-protein mass ratio for the first digestion overnight and a 1:100 trypsin-to-protein mass ratio for a second 4 h digestion. After trypsin digestion, the peptide was desalted by a Strata X C18 SPE column (Phenomenex, Tianjin, China) and vacuum-dried. The peptide was reconstituted in 0.5 M TEAB and processed according to the manufacturer’s protocol for the iTRAQ kit (Sigma-Aldrich, Shanghai, China). Briefly, one unit of iTRAQ reagent was thawed and reconstituted in acetonitrile. The peptide mixtures were then incubated for 2 h at room temperature and pooled, desalted and dried by vacuum centrifugation. The tryptic peptides were fractionated by high pH reverse-phase HPLC using an Agilent 300Extend C18 column (5 μm particles, 4.6 mm ID, 250 mm length). Briefly, peptides were first separated with a gradient of 8% to 32% acetonitrile (pH 9.0) over 60 min into 60 fractions. Then, the peptides were combined into 18 fractions and dried by vacuum centrifugation. The tryptic peptides were dissolved in 0.1% formic acid (solvent A) and loaded directly onto a homemade reversed-phase analytical column (15 cm length, 75 μm i.d.). The gradient comprised an increase from 6 to 23% solvent B (0.1% formic acid in 98% acetonitrile) over 26 min, 23% to 35% in 8 min, 35% to 80% in 3 min and then a hold at 80% for the last 3 min, all at a constant flow rate of 400 nL/min on an EASY-nLC 1000 UPLC system. The peptides were subjected to an NSI source followed by tandem mass spectrometry (MS/MS) in a Q ExactiveTM Plus (Thermo Fisher Scientific, Shanghai, China) coupled online to UPLC. The electrospray voltage applied was 2.0 kV. The m/z scan range was 350 to 1800 for the full scan, and intact peptides were detected in the Orbitrap at a resolution of 70,000. Peptides were then selected for MS/MS using the NCE setting of 28, and the fragments were detected in the Orbitrap at a resolution of 17,500. A data-dependent procedure alternated between one MS scan followed by 20 MS/MS scans with 15.0 s dynamic exclusion. The automatic gain control (AGC) was set at 5E4. The fixed first mass was set as 100 m/z. Proteome Discover 2.4 (Thermo Fisher Scientific, Shanghai, China) was used for data processing. Tandem mass spectra were searched against the UniProt-reviewed Homo sapiens (Human) database concatenated with the reverse decoy database. Trypsin/P was specified as a cleavage enzyme allowing up to 2 missing cleavages. The mass tolerance for precursor ions was set as 20 ppm in the first search and 5 ppm in the main search, and the mass tolerance for fragment ions was set as 0.02. Carbamidomethyl on Cys was specified as a fixed modification, and oxidation on Met was specified as a variable modification. FDR was adjusted to < 1%, and the minimum score for peptides was set at 40. The identification of proteins and screening of differentially expressed proteins were performed. The threshold for differentially expressed proteins was set as *P* < 0.05 and fold changes of ≥ 1.2-fold or ≤ 0.83-fold. Differentially expressed proteins were further analyzed by principal component analysis (PCA) and Gene Ontology (GO), Kyoto Encyclopedia of Genes and Genomes (KEGG) and STRING database analyses. The mass spectrometry proteomics data have been deposited in the ProteomeXchange Consortium (http://proteomecentral.proteomexchange.org) via the iProX partner repository with the dataset identifier PXD022209.

### *Cell culture and *in vitro* decidualization*

Immortal human ESCs (T-HESC) were cultured in phenol red-free DMEM/F12 (Gibco, New York, USA) containing 10% charcoal/dextran-treated FBS (Gibco, New York, USA), 100 IU/ml penicillin (Beyotime, Shanghai, China), and 100 μg/ml streptomycin (Beyotime, Shanghai, China) at 37 °C under 5% CO_2_ humidified air. Treatment included 0.5 mM 8-bromo-cAMP (MedChemExpress, Shanghai, China) and 1 μM medroxyprogesterone acetate (MPA) (MedChemExpress, Shanghai, China). In vitro artificially induced decidualization was achieved as previously described [17]. For decidualization experiments, confluent T-HESC monolayers were decreased in 2% DMEM/F12 media (DMEM/F12 media without phenol red, 2% FBS) overnight before treatment with 0.5 mM 8-bromo-cAMP and 1 μM medroxyprogesterone acetate (MPA). T-HESCs were induced for in vitro decidualization for 4 days using the same method described above, and the culture medium was changed every 2 days. Enzymatic activity of TOP2A was altered by treatment with 1 μM Etoposide (MedChemExpress, Shanghai, China) for 48 h.

### Lentivirus transfection

Lentiviruses carrying short hairpin RNA (shRNA) targeting human TOP2A were purchased from GeneChem (Shanghai, China). The sequences for the TOP2A knockdown virus are listed below: sh-TOP2A#1 (5′- gcTCCAAATCAATATGTGATT -3′), sh-TOP2A#2 (5′-gcCTGATTTGTCTAAGTTTAA-3′), sh-TOP2A#3 (5′-ccCAACTTTGATGTGCGTGAA-3′), and scrambled control shRNA (5′‐TTCTCCGAACGTGTCACGT‐3′). The lentiviral complex infection index (MOI) was determined by preliminary experiments to be 20. For lentivirus transfection, when the cells reached 20–30% confluence, T-HESCs were transfected with lentivirus and HiTransG lentivirus infection reagent (GeneChem, Shanghai, China) for 48 h according to the manufacturer's protocol. Two days after transfection, the transfected cells were screened using 2 μg/ml puromycin (Beyotime, Shanghai, China) for one week to establish stably transfected cell clones. Knockdown of TOP2A was verified by Western blot analysis and qRT-PCR.

### Immunohistochemistry staining

Human endometrial tissues were washed with PBS, fixed overnight with 4% paraformaldehyde at room temperature and sectioned into 5 μm-thick sections after being dehydrated and embedded in paraffin. For immunohistochemistry, the tissue sections were deparaffinized and rehydrated in a graded alcohol series. Antigen retrieval was achieved by a microwaved pretreatment in 10 mM citric sodium (pH: 6.0) for 15 min. Then, sections were blocked with 3% H_2_O_2_ at room temperature for 15 min for endogenous peroxidase ablation. Then, the sections were incubated with a mouse mAb against TOP2A (1:250; Proteintech, Rosemont, USA) at 4 °C overnight. The next day, a secondary antibody conjugated with horseradish peroxidase was applied for 30 min at RT. The immunocomplex was then visualized by diaminobenzidine. Images were captured using an Olympus VS200 ASW 3.2.1 system.

### Western blot

Total protein was extracted from the tissues or cells using RIPA lysis buffer (Beyotime, Shanghai, China) with phenylmethanesulfonyl fluoride (PMSF) (Beyotime, Shanghai, China), and the protein concentration was determined by a BCA protein assay kit (Beyotime, Shanghai, China). The lysates were separated by SDS-PAGE (Bio-Rad Laboratories, California, USA) and transferred to PVDF membranes (Millipore Sigma, Massachusetts, USA). After the membranes were blocked with 5% nonfat dry milk (Bio-Rad Laboratories, California, USA) at room temperature for 1 h, they were then incubated overnight at 4 °C with antibodies against the proteins of interest, including TOP2A (1:10,000; ab52934, Abcam, Shanghai, China), IGFBP1 (1:1000; ab181141, Abcam, Shanghai, China), p65 (1:1000; ab32536, Abcam, Shanghai, China), p-p65 (1:1000; ab183559, Abcam, Shanghai, China), IKK (1:1000; #2682, Cell Signaling Technology, Shanghai, China), p-IKK (1:1000; #2697, Cell Signaling Technology, Shanghai, China), and β-actin (1:1000; 2D4H5, Proteintech, Rosemont, USA), overnight at 4 °C. The PVDF membranes were washed and then incubated with horseradish peroxidase-conjugated secondary antibodies (1:5000, ZSGB-BIO, Shanghai, China) at RT for 1 h. Protein blots were visualized by enhanced chemiluminescent reagents (Millipore Sigma, Massachusetts, USA), and pictures were captured by a Vilber Fusion image system (Fusion FX5 Spectra, Marne-la-Vallée cedex 3, France).

### Real-time quantitative PCR

Total RNA was extracted from T-HESCs, endometrial tissues and uterine tissues using TRIzol reagent (Invitrogen, Shanghai, China). The RNA concentration was measured by ultraviolet spectroscopy (NanoDrop2000, Thermo Fisher Scientific, MA, USA). The Prime Script RT reagent Kit (Roche Life Science, Shanghai, China) was used to synthesize cDNA following the manufacturer’s instructions. SYBR Green dye (Roche Life Science, Shanghai, China) was used to perform real-time PCR in an Applied Biosystems PCR cycler. The PCR primer sequences are listed below. The mean threshold cycle (Ct) values were normalized to those of β-actin, and the relative mRNA levels of TOP2A, IGFBP1 and PRL were analyzed.

**Table:** 

Primers (human)	Sequences (5' → 3')
TOP2A	Forward TAATCAGGCTCGCTTTATCTT
	Reverse TCCGAATCATATCCCCTCT
IGFBP1	Forward CTATGATGGCTCGAAGGCTC
	Reverse TTCTTGTTGCAGTTTGGCAG
PRL	Forward TCTCGCCTTTCTGCTTATTATAAC
	Reverse CGATTCGGCACTTCAGGAGCTT
β-actin	Forward TGGCACCCAGCACAATGAA
	Reverse CTAAGTCATAGTCCGCCTAGAAGCA
Primers (mouse)	Sequences (5' → 3')
TOP2A	Forward ACAGGTGGTCGAAATGGCTA
	Reverse AGGGCTTGAGTTCCATGTCA
β-actin	Forward TGTGACGTTGACATCCGTAAAG
	Reverse TCAGTAACAGTCCGCCTAGAA

### Immunofluorescence staining

T-HESCs were grown in 6-well cell culture plates. After three 5 min washes with PBS, the T-HESCs were fixed with 4% paraformaldehyde (PFA) in phosphate-buffered saline (PBS) at room temperature for 30 min. After the cells were washed with PBS, they were permeabilized with 0.2% Triton X-100 in PBS for 5 min at room temperature and blocked with 5% bovine serum albumin (BSA) for 1 h at RT. Filamentous actin was visualized by CytoPainter phalloidin-iFluor 488 Reagent (1:1000; ab176753, Abcam, Shanghai, China). NF-κB was determined using an anti-p65 (1:500; ab32536, Abcam, Shanghai, China) primary antibody. After three washes with BSA/PBS, the cells were further incubated with Cy3–conjugated AffiniPure goat anti-rabbit IgG (1:100, Proteintech, Rosemont, USA). Nuclei were stained with 4′,6-diamidino-2-phenylindole dihydrochloride (DAPI) (Sigma-Aldrich, Shanghai, China). Finally, staining was visualized by light microscopy, and images were captured.

### mRNA sequencing

Negative control and stable sh-TOP2A T-HESCs treated with both 8-bromo-cAMP and MPA for 4 days were collected for total RNA extraction (Invitrogen, Shanghai, China). Total RNA was tested by agarose gel electrophoresis for quality control. The purity and integrity of RNA were assessed by a SMA4000 spectrophotometer (Meriton, Beijing, China) and Agilent 2100 Bioanalyzer (Agilent Technologies, US), respectively. A total of 1 μg of RNA from each sample served as input material for the RNA sample preparations. The library was validated on a Bioptic Qsep100 Analyzer (Bioptic, Inc., Taiwan, China). The products were amplified and sequenced using Illumina HiSeq2500. Differential expression analysis was performed using the DESeq2 R package. An adjusted *P* value of 0.025 and absolute fold change of 2 served as the thresholds for significant differential expression. Differentially expressed genes were further analyzed by Gene Ontology (GO) and Kyoto Encyclopedia of Genes and Genomes (KEGG) database analyses.

### Establishment of TOP2A-deficient mice

All experimental procedures involving animals were performed in compliance with the Guidelines for the Care and Use of Laboratory Animals, approved by the Laboratory Animal Welfare and Ethics Committee of Chongqing Medical University, and reported in accordance with the ARRIVE (Animal Research: Reporting of In Vivo Experiments) guidelines. Eight to 12-week-old C57BL/6 J virgin female mice were bred with age‐matched males. The mice were housed in a temperature-controlled room under specific-pathogen-free conditions and a standard 12-h light/dark cycle, with ad libitum access to food and water. The day on which the vaginal plug was detected was considered GD0.5. The pregnant mice were randomly assigned to 2 groups: adenovirus (ad)-control (Ctrl) and ad-TOP2A (*n* = 6 for both groups). GFP-tagged TOP2A-interfering adenovirus was purchased from TsingKe Biotech, Beijing, China. On GD2.5 and GD3.5, adenovirus was injected into pregnant mice via the tail vein (2 × 10^8^ PFU, 100 μl). The shRNA sequence used to target TOP2A was 5′-GCAGACTACATTGCCGTTTAA-3′. All dams from each group were sacrificed on GD8.5.

### Statistical analyses

The data are presented as the mean ± SEM. Statistical data were analyzed by the Mann–Whitney U test or one-way ANOVA. A value of *p* < 0.05 was considered significant. The statistical analyses were performed using Prism 8 software (GraphPad Software, La Jolla, CA, USA).

## Results

### Clinical features of patients with RIF

The distribution of demographic characteristics and clinical characteristics of the study subjects are summarized in Table 1. As shown in Table 1, no significant differences in age, BMI, or basal FSH, LH or estradiol levels were found between the RIF and control groups except for the number of embryo transfers.

**Table 1:** Clinical characteristics of women enrolled in the present study

Variables	Normal groups(*n*=12)	RIF patients(*n*=12)	Proliferative phase (*n*=12)	Mid-secretory phase (*n*=12)	*p*-value
Age (y)	30.92±2.575	30.83±3.433	30.25±3.223	31.37±2.823	0.8983^a^
BMI (kg/m^2^)	22.25±3.191	20.66±2.292	22.39±2.014	21.18±2.827	0.3102^a^
FSH (mIU/ml)	7.611±0.7842	7.456±0.9484	7.151±1.711	7.134±1.443	0.7505^a^
LH (mIU/ml)	5.586±0.8098	5.331±0.7227	4.347±1.575	5.145±1.435	0.0802^a^
Estradiol (pg/ml)	31.99±10.87	34.17±10.42	36.30±16.55	37.76±9.392	0.6727^a^
Number of embryo transfer	1 (9/12 75%)2 (3/12 25%)	4(5/12 41.67%)5(7/12 58.33%)	/	/	<0.001^b^

^a^One-way ANOVA. ^b^Fisher’s exact test. BMI: body mass index.

These continuous data were expressed as mean ± SD. Differences with *P*<0.05 were considered statistically significant

#### Proteomic analysis indicates that TOP2A might be a pivotal molecule in RIF

**Fig. 1:**
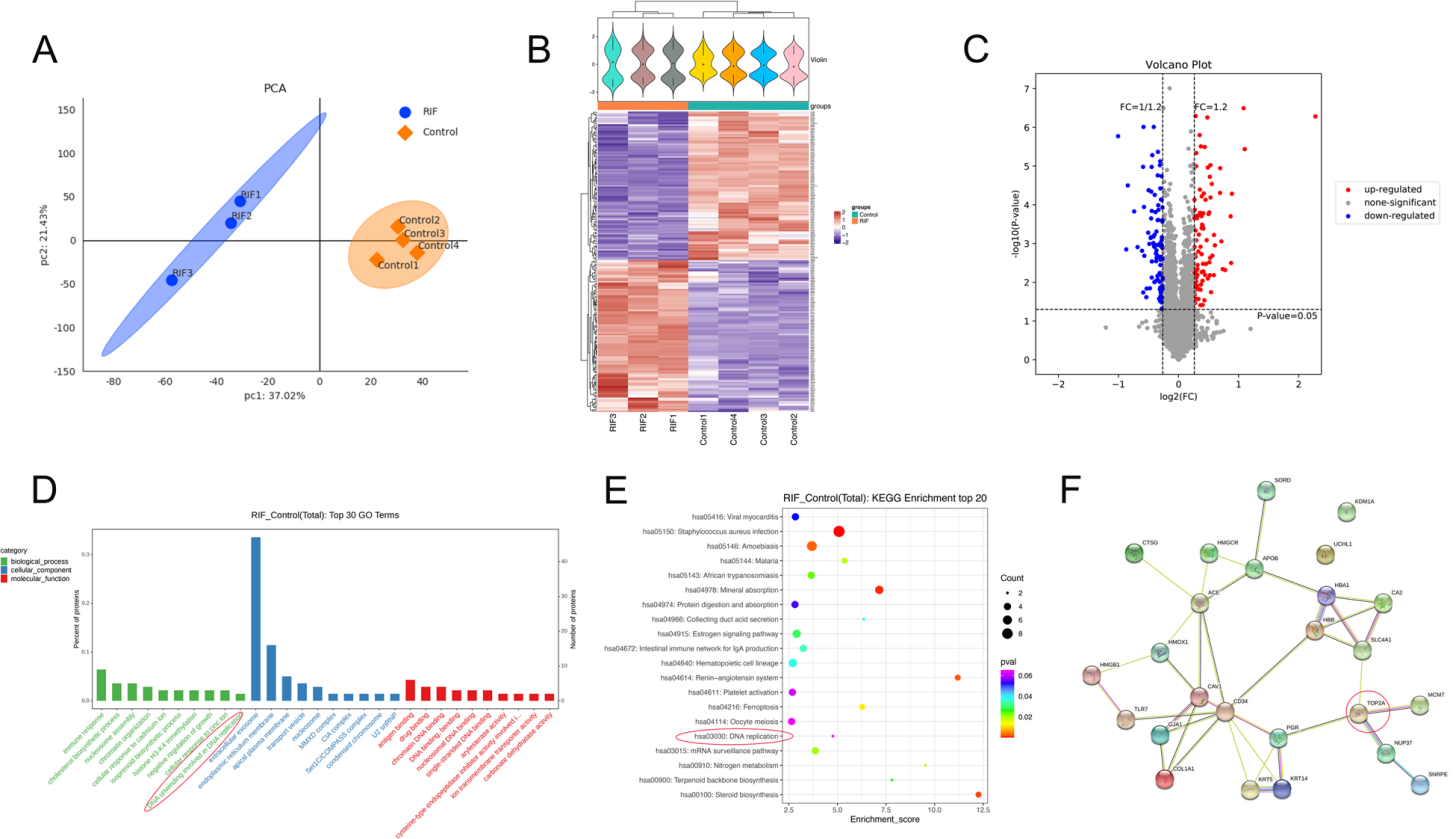
Proteomic analysis comparing the recurrent implantation failure (RIF) and control groups. **A** Two-dimensional scatter plot of principal component analysis (PCA) results. Samples in the control group (*n* = 4) and the RIF group (*n* = 3) are represented by blue dots and orange dots, respectively. **B** Hierarchical clustering of differentially expressed proteins. Rows in the heatmap correspond to differentially expressed proteins, and columns correspond to samples. Samples in the control group and the RIF group are highlighted in blue and orange, respectively. **C** Volcano plot of all expressed proteins. Differentially expressed proteins with upregulated and downregulated expression with *p* < 0.05 and fold change ≥ 1.2 are highlighted in blue and red, respectively. **D** Functional enrichment results of differentially expressed proteins, including GO category: biological process, cellular components and molecular function. The x-axis represents GO terms, and the y-axis represents the number and percent of differentially expressed proteins. GO terms related to DNA unwinding involved in DNA replication are highlighted in red circles. **E** KEGG pathway analysis showed that the top 20 ranking pathways related to DNA replication are highlighted in red circles. **F** The PPI network of the top 25 proteins in connectivity was constructed using Cytoscape. TOP2A is highlighted in red circle

Comparative proteomic analysis was performed on 3 RIF and 4 control endometrial tissues. Principal component analysis comparing all patients with RIF to the controls revealed that the RIF group was accurately separated from the control group in 2 dimensions (Fig. 1A). All differentially expressed proteins were clustered using a hierarchical clustering algorithm (Fig. 1B), and the protein expression pattern of the RIF group was significantly different from that of the control group. Of the 188 differentially expressed proteins, 95 had upregulated expression and 93 had downregulated expression (Fig. 1C), and TOP2A was included in the decreased proteins. Gene Ontology (GO) and Kyoto Encyclopedia of Genes and Genomes (KEGG) pathway analysis clustered these differentially expressed proteins into groups based on their biological processes, molecular functions and cellular components (Fig. 1D, E). Notably, ‘DNA replication’ and ‘DNA unwinding involved in DNA replication’ comprised the majority of the enriched KEGG pathway and GO terms. In particular, the level of TOP2A, a representative protein that regulates DNA replication, was significantly decreased in the RIF group compared with the control group. Using the STRING database to analyze protein interactions with the differentially expressed proteins, we selected the top 25 proteins in terms of connectivity and generated an interaction network diagram, and we found that TOP2A is an important protein in this network (Fig. 1F).

#### Decreased expression of TOP2A in the patients with RIF

**Fig. 2:**
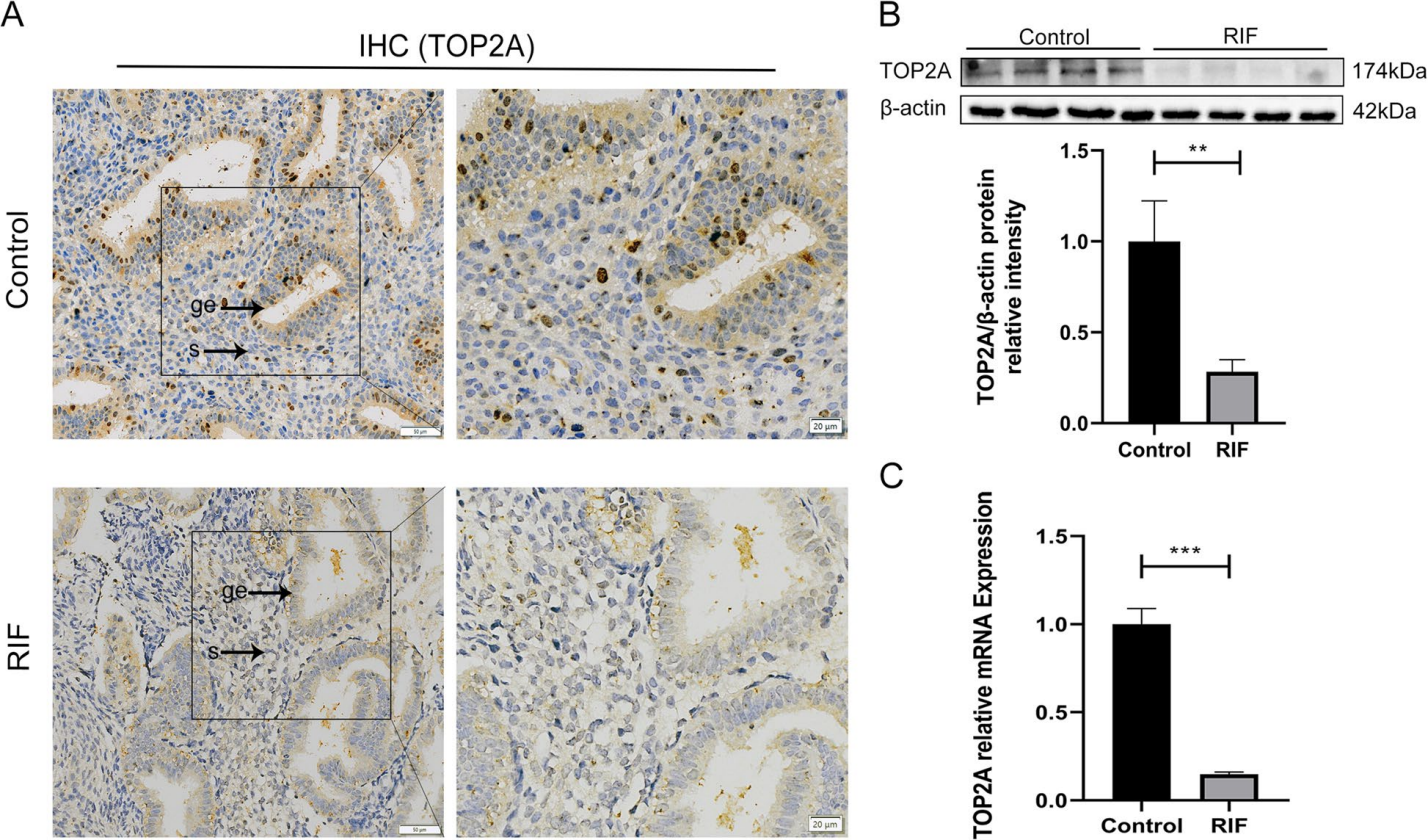
Decreased expression of TOP2A in the endometria of patients with RIF. **A** Immunohistochemistry analysis of paraffin-embedded endometrial tissues. TOP2A expression in the endometria of the control women and the patients with RIF. Immunohistochemical analysis was performed using a TOP2A antibody. Original magnification left panel: × 100, scale bar = 50 μm, right panel: × 200, scale bar = 20 μm. s, stroma; ge, glandular epithelium. **B**, **C** The protein and mRNA expression of TOP2A were examined separately by Western blotting and RT-qPCR in human endometria, *n = *12 in each group. Representative Western blotting results for the control group and the RIF group are shown. Mann–Whitney U test. All data are represented as the mean ± SEM. **p < 0.01, ***p < 0.001

Immunohistochemistry analysis showed that TOP2A was localized in both stromal and glandular epithelial cells of the endometrium (Fig. 2A). TOP2A staining was weaker in the RIF group, consistent with the Western blotting results. The TOP2A protein expression was tested in the patients with RIF and the controls by Western blotting. The patients with RIF showed a significant decrease in the protein expression level of TOP2A compared with the controls (Fig. 2B), which is consistent with the mRNA results shown below (Fig. 2C).

#### TOP2A expression is downregulated under stimulation by 8-bromo-cAMP and MPA

**Fig. 3:**
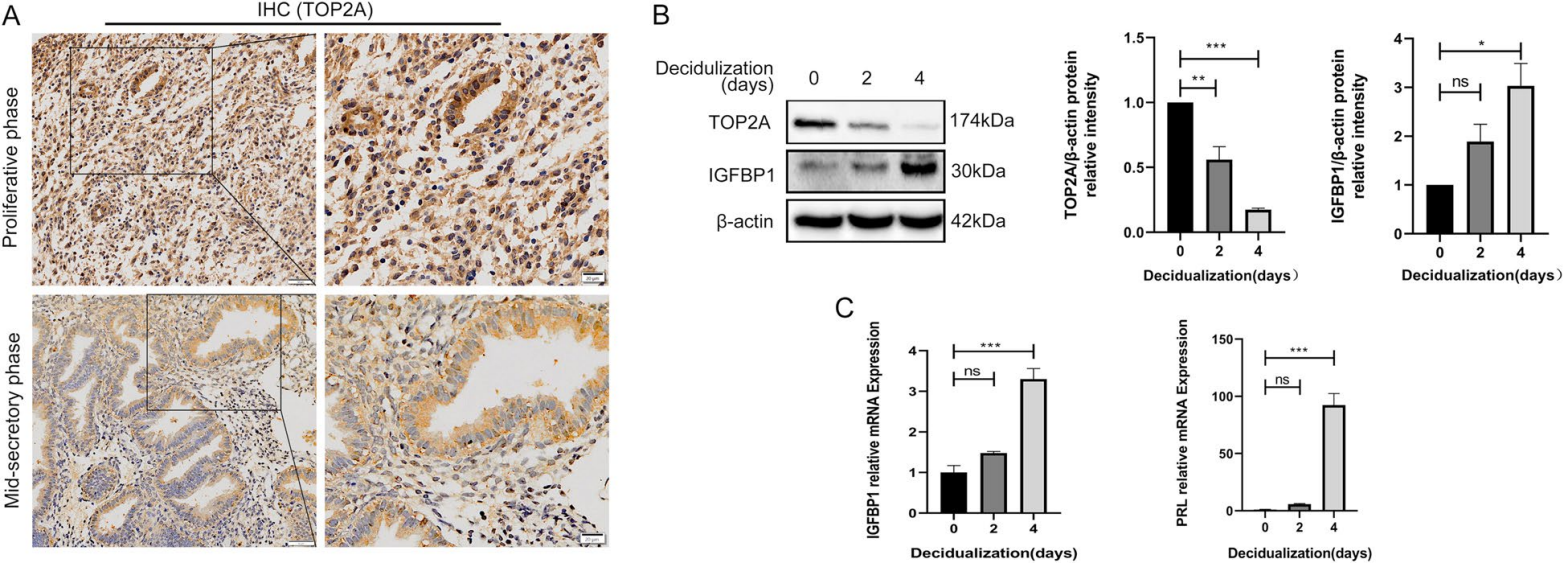
TOP2A expression pattern in human endometrial tissue; the level of TOP2A in T-HESCs decreases upon decidualization. **A** Immunohistochemistry analysis of paraffin-embedded endometrial tissues in the proliferative and mid-secretory phases. Original magnification left panel: × 100, scale bar = 50 μm, right panel: × 200, scale bar = 20 μm. *n* = 3 in each group. **B**, **C** TOP2A and IGFBP1 protein levels and IGFBP1 and PRL mRNA expression in T-HESCs were measured by Western blotting and RT-qPCR every 48 h over a 4-day decidualization time course. Data are the mean ± SEM of 3 biological replicates unless stated otherwise. One-way ANOVA. *p < 0.05, **p < 0.01, ***p < 0.001 compared to Day 0. ns, nonsignificant

To evaluate the functional role of TOP2A in embryo implantation, we examined TOP2A expression during different phases of the menstrual cycle. An immunolocalization analysis suggested that the TOP2A protein abundance in glandular epithelial and stromal cells of the endometrium was lower at the mid-secretory phase of the menstrual cycle (Fig. 3A). Immortal human ESCs (T-HESCs) were employed to examine the role of TOP2A in decidualization with both 8-bromo-cAMP and MPA in vitro. The results showed that TOP2A expression was significantly downregulated after 4 days of stimulation with 8-bromo-cAMP and MPA, while the expression of the decidual markers IGFBP1 and PRL was significantly increased (Fig. 3B, C).

#### *TOP2A deficiency enhances T-HESC decidualization *in vitro

**Fig. 4:**
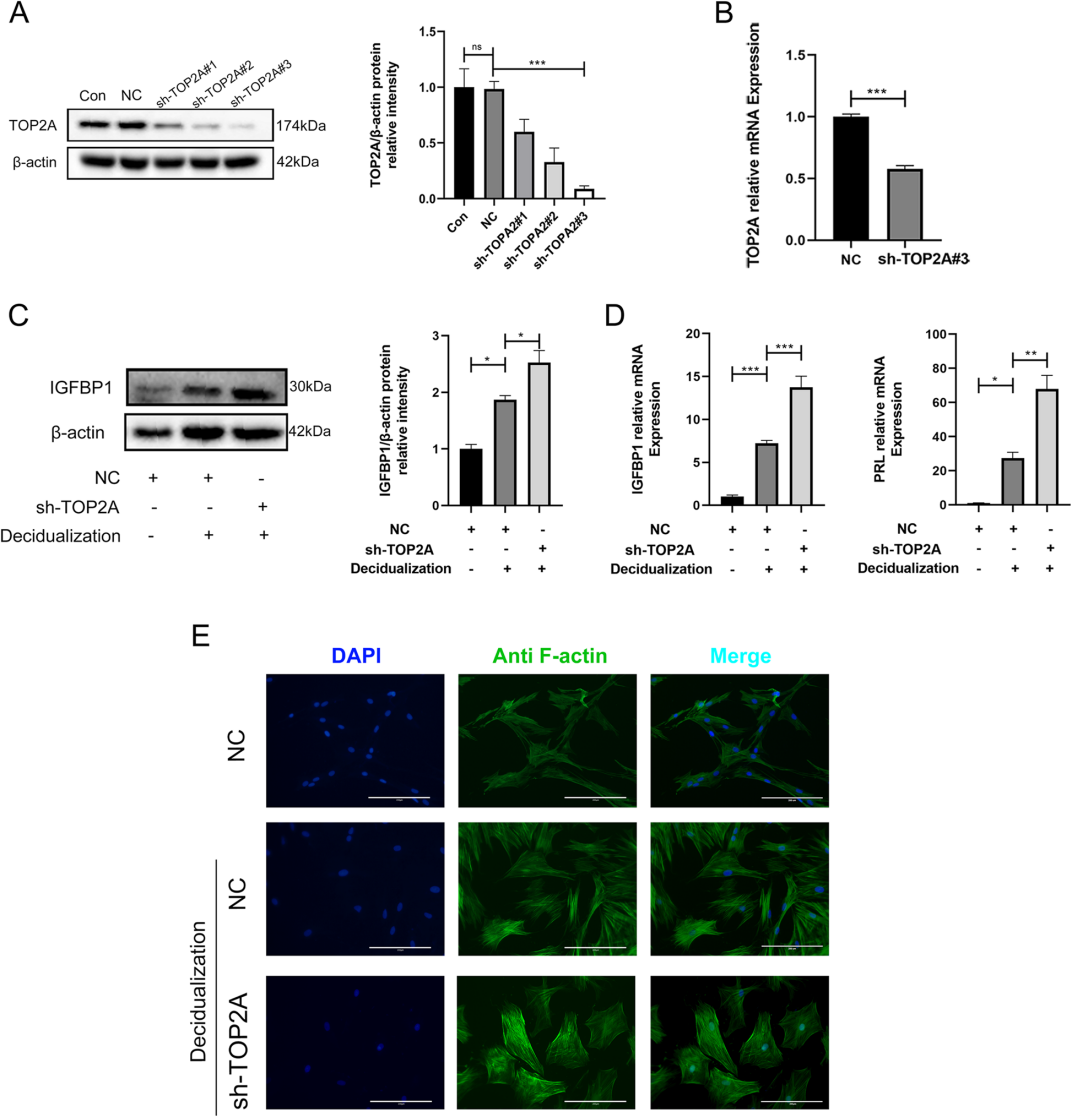
Loss of TOP2A enhances decidualization in T-HESCs. **A** Western blotting of TOP2A in T-HESCs. Con, untreated cells; NC, negative control cells transfected with scramble shRNA; sh-TOP2A#1–3, cells transfected with different shRNAs targeting TOP2A. **B** The mRNA expression of TOP2A in the NC and sh-TOP2A#3 T-HESCs was measured by RT-qPCR. **C** IGFBP1 protein expression in T-HESCs following transfection with NC or sh-TOP2A. The cultures either remained untreated or were decidualized for 4 days. **D** IGFBP1 and PRL mRNA expression in T-HESCs following transfection with NC or sh-TOP2A. The cultures either remained untreated or were decidualized for 4 days. **E** CytoPainter phalloidin-iFluor 488 reagent was used to label filamentous actin, and immunofluorescence was used to analyze the morphological transformation of T-HESCs. Decidualized cells were treated with both 8-bromo-cAMP and MPA for 4 days. (F-actin, green fluorescence signals; DAPI, blue signals; scale bar = 200 μm). One-way ANOVA or Mann–Whitney U test. Data are the mean ± SEM of 3 biological replicates unless stated otherwise. *p < 0.05, **p < 0.01, ***p < 0.001. ns, nonsignificant

To investigate whether TOP2A is a determinant of decidualization in embryo implantation, we generated TOP2A knockdown (sh-TOP2A#3) T-HESCs, in which the expression levels of TOP2A were repressed by approximately 80% (Fig. 4A, B). The T-HESCs with TOP2A knockdown showed significantly increased IGFBP1 protein and IGFBP1 and PRL mRNA expression levels (Fig. 4C, D). In decidualized T-HESCs, filamentous actin (F-actin) staining showed the expected cytoskeletal reorganization and shape changes consistent with the transformation from a fibroblastoid to decidual phenotype. The knockdown of TOP2A strengthened the characteristic phenotypic modification during decidualization (Fig. 4E). Meanwhile, we treated the T-HESEs with Etoposide, a TOP2 inhibitor, to inhibit the enzymatic activity of TOP2A. Under the treatment with Etoposide, the expression level of TOP2A was decreased, the IGFBP1 and PRL expression levels were accelerated. Immunofluorescence showed the inhibition of TOP2A strengthened the actin cytoskeleton reorganization (Fig. S2).

#### The NF-κB signaling pathway plays a role in the regulation of decidualization

**Fig. 5:**
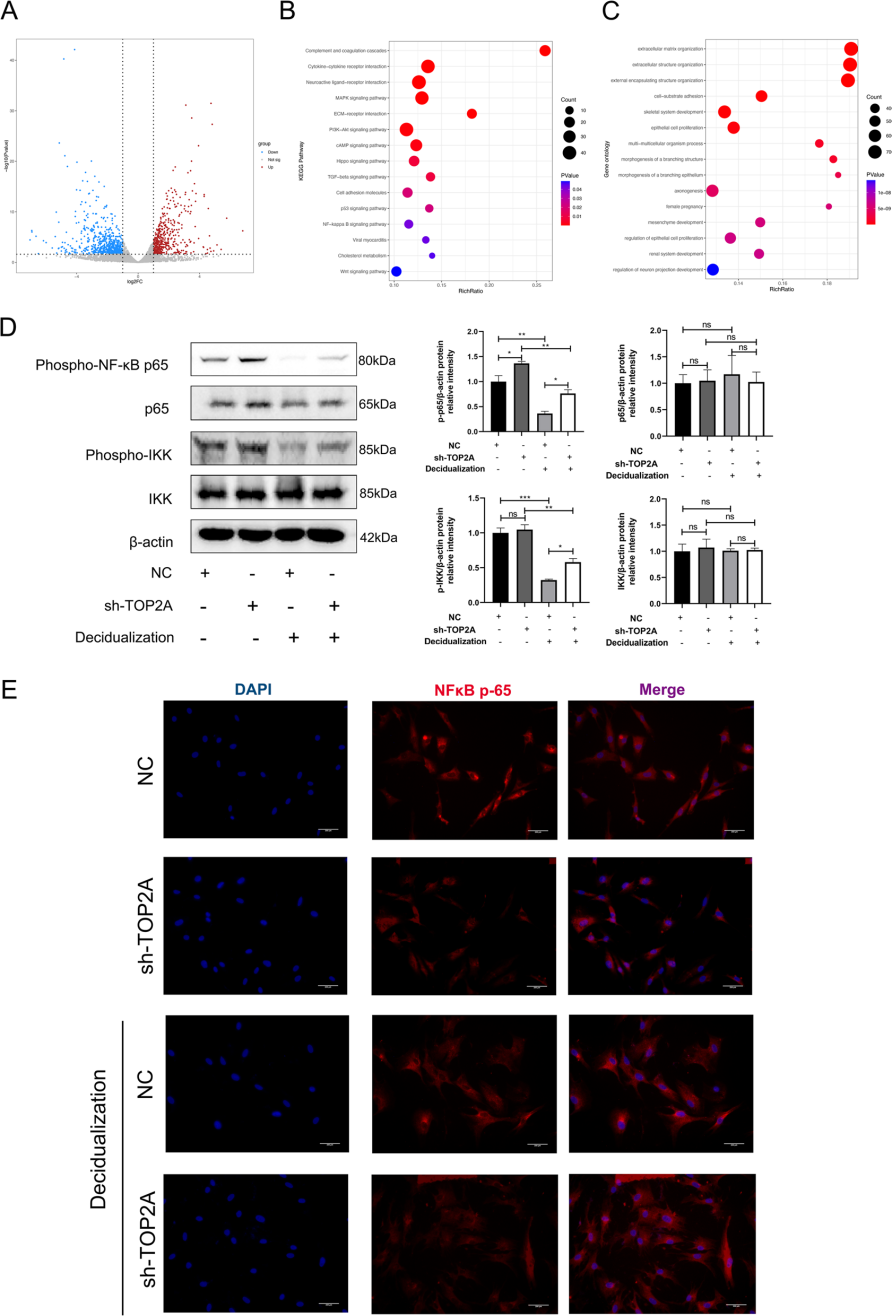
TOP2A regulates decidualization through the NF-κB signaling pathway in T-HESCs. **A** Volcano plot of the DEGs between the sh-TOP2A and NC T-HESCs treated with both 8-bromo-cAMP and MPA for 4 days; the DEGs were analyzed by (**B**) KEGG and (**C**) GO analysis. **D** Protein levels of p-p65, p65, IKK and p-IKK were examined separately by Western blotting in various T-HESCs. **E** Effect of TOP2A on the nuclear translocation of NF-κB p65. Decidualized cells were treated with both 8-bromo-cAMP and MPA for 4 days. The nuclear translocation of p65 was evaluated by immunofluorescence staining (p65, red fluorescence signals; DAPI, blue signals; scale bar = 200 μm). One-way ANOVA. Data are the mean ± SEM of 3 biological replicates unless stated otherwise. *p < 0.05, **p < 0.01, ***p < 0.001. ns, nonsignificant

To elucidate the mechanism underlying TOP2A deficiency-enhanced decidualization in T-HESCs, we subjected the decidualized sh-TOP2A(sh-TOP2A-de) and NC (NC -de) T-HESCs to mRNA sequencing. Compared with the NC-de T-HESCs, the sh-TOP2A-de T-HESCs showed significant alterations in the expression levels of 1187 mRNAs (463 with upregulated and 724 with downregulated expression, Fig. 5A). The differentially expressed genes (DEGs) were further enriched based on the KEGG and GO analyses (Fig. 5B, C). Notably, the NF-κB signaling pathway was enriched (Fig. 5B), and the DEGs were involved in cell proliferation, as indicated by GO analysis (Fig. 5C). Since the NF-κB signaling pathway may be involved in regulating decidualization [18, 19], we further explored the effects of TOP2A on the NF-κB signaling pathway by using Western blotting to detect IκB kinase (IKK) and NF-κB p65 (p65) phosphorylation levels. Our results revealed that TOP2A knockdown increased phospho-NF-κB p65 (p-p65) protein levels. Although p-p65/p-IKK was decreased after decidualization, the expression of p-p65/p-IKK in decidualization was increased in the TOP2A knockdown cells compared with the control cells (Fig. 5D). We investigated the translocation of p65 from the cytoplasm into the nucleus using an immunofluorescence assay. After decidualization, the nuclear translocation of p65 was weakened, as shown by the attenuation of p65 fluorescence staining in the nucleus. However, in decidualized T-HESCs, TOP2A knockdown resulted in enhanced p65 fluorescence staining in the nucleus, indicating increased nuclear translocation of p65 (Fig. 5E).

#### Lack of TOP2A predisposes mice to impaired fertility

**Fig. 6:**
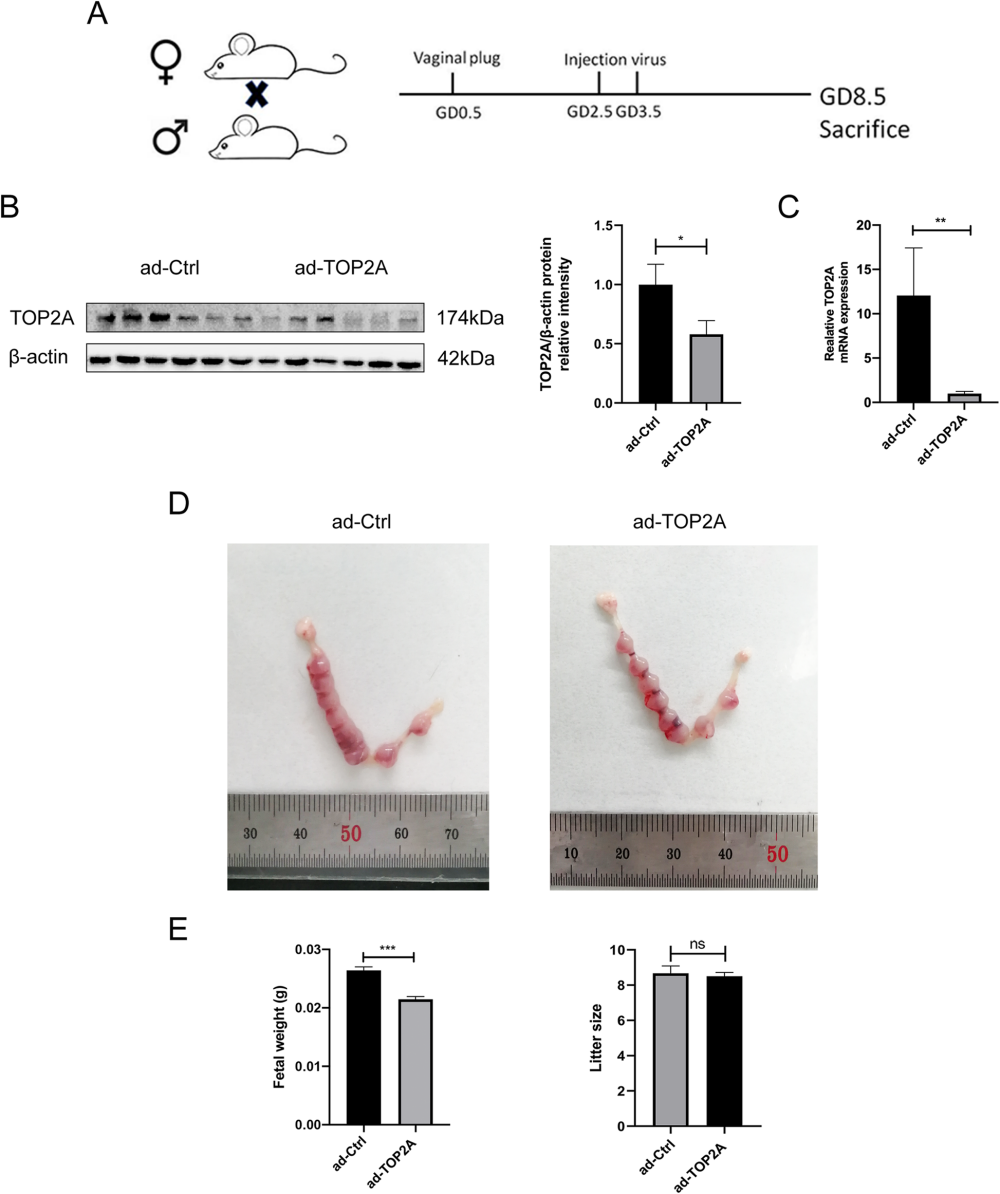
TOP2A deficiency leads to impaired fertility in mice. **A** Flow diagram of the experimental design. **B** Western blotting of TOP2A protein expression in the mouse uterus at GD8.5 (ad-Ctrl, *n* = 6; ad-TOP2A, *n* = 6). **C** RT-qPCR of TOP2A mRNA expression in the mouse uterus at GD8.5 (ad-Ctrl, *n* = 6; ad-TOP2A, *n* = 6). **D** Representative images of the mouse uterus at GD8.5 (ad-Ctrl, *n* = 6; ad-TOP2A, *n* = 6). **E** Mice were sacrificed on GD8.5, and the fetuses were dissected from the uterus and weighed. Litter size and fetal weight were recorded (ad-Ctrl, *n* = 52 pups from 6 dams; ad-TOP2A, *n = *51 pups from 6 dams). Mann–Whitney U test. Data are the mean ± SEM of 3 biological replicates unless stated otherwise. *p < 0.05, **p < 0.01, ***p < 0.001. ns, nonsignificant

To verify our findings from in vitro studies, we generated TOP2A-deficient mice (ad-TOP2A mice) by injection of TOP2A-interfering adenovirus through the tail vein on GD2.5 and GD3.5. The experimental design is shown in Fig. 6A. A significant reduction in uterine TOP2A protein and mRNA expression was observed on GD8.5 (Fig. 4B, C). Compared to that in the control group, the litter size of the ad-TOP2A mice was decreased, but the difference was not significant. However, the fetal weights recorded on GD8.5 were significantly decreased in the ad-TOP2A mice (Fig. 6D, E).

## Discussion

IVF results in several thousand successful pregnancies each year. Rapid changes in IVF practices and technologies have occurred since their introduction. In recent years, with the advent of vitrification and improved IVF cycle outcomes after blastocyst transfer [20], successful implantation has been found to require a receptive endometrium, a normal and functional embryo at the blastocyst developmental stage and a synchronized dialog between maternal and embryonic tissues [21, 22]. In this case, the focus of implantation failure shifts from the embryo to the endometrium. Some studies have suggested that inadequate uterine receptivity is responsible for approximately two-thirds of implantation failures, whereas the embryo itself is responsible for only one-third of these failures [5, 23, 24]. To explore the potential molecular mechanism of uterine receptivity failure, we obtained biopsies at the mid-secretory stage, especially the day of blastocyst transfer, and investigated the differentially expressed proteins in patients with RIF and normal fertile controls. According to the GO and KEGG analyses, various proteins associated with DNA replication showed abnormal changes. In particular, hierarchical cluster analysis revealed that the TOP2A protein exhibited a significant change. However, few studies have investigated the role of TOP2A in the endometrium and in embryo implantation.

The human endometrium is a unique tissue that proliferates, differentiates, and sloughs off during the menstrual cycle. This complex transformation allows the endometrium to perform a variety of multifunctional tasks. The establishment of endometrial receptivity is primarily coordinated by estrogen and progesterone through direct and indirect transcriptional and translational regulation, leading to changes in the function of all endometrial cell types [25, 26]. The endometrium maintains complex control of proliferation and apoptosis as part of each menstrual cycle that prepares the endometrium for the window of implantation and pregnancy [27]. Estrogen stimulates endometrial proliferation, with the resulting thickness directly correlated with the success of assisted reproduction [28]. In this study, for the first time, we found that the expression level of TOP2A was downregulated in the mid-secretory phase endometrium of women with RIF by proteomic analysis. We also showed that the expression of TOP2A periodically decreased during the menstrual cycle. We further showed that TOP2A is expressed in both endometrial glandular epithelial cells and stromal cells in endometrial tissues and that the level of TOP2A expression in stromal cells was significantly decreased in the RIF group compared with the control group. TOP2A is a cell cycle-dependent protein whose expression is highly dependent on cell proliferation and can be suppressed when cell proliferation and division stop [29–31]. Studies have also found that TOP2A is an oncogene. TOP2A is expressed at a moderate level in normal tissues and cells. However, it is highly expressed in ovarian cancer and breast cancer, and its expression is closely related to tumor progression, recurrence and poor prognosis [32, 33]. Bassi MA et al. found that the expression of TOP2A in the endometrium is decreased in patients with endometriosis [34]. Therefore, we speculate that TOP2A may lead to implantation failure by affecting the proliferation and differentiation of endometrial stromal cells in patients with RIF. In most studies [26, 35], endometrial decidualization is an important point for the study of endometrial receptivity. Next, we explored the relationship between TOP2A and decidualization.

In differentiating human endometrial stromal cells (HESCs), cAMP and progesterone signaling converge on forkhead box O1A (FOXO1), a core decidual transcription factor responsible for cell cycle arrest and the induction of decidual marker genes, such as PRL and IGFBP1 [36]. To gain insight into the mechanism that drives decidualization, we measured TOP2A expression in the cultured T-HESCs treated for 4 days with a combination of 8-bromo-cAMP and MPA. We showed that TOP2A expression gradually decreased in response to 8-bromo-cAMP and MPA treatment in a time-dependent manner. However, the lentivirus-mediated knockdown of TOP2A markedly enhanced decidual PRL and IGFBP1 expression. Decidualization of the T-HESCs involves notable morphological and functional differentiation, which is a crucial step in making the endometrium receptive to an embryo [37]. Knockdown of TOP2A enhanced the cytoskeletal structure of decidualizing T-HESCs, such that TOP2A could be implicated in the regulation of the decidualization process, which depends on actin cytoskeleton reorganization [38]. To make this result more credible, we treated the T-HESEs with Etoposide and obtained consistent results (Fig. S2). These results suggest that the deficiency of TOP2A signaling is a crucial step contributing to the pathogenesis of RIF by accelerating the process of decidualization. These results were unexpected. In the patients with RIF, the expression of TOP2A decreased, but silencing TOP2A did not impair decidualization, as expected; in contrast, it enhanced decidualization. These results showed that in the receptive endometrial state, decidualization needs to achieve a balance. Excessive or insufficient decidualization will cause implantation failure. Similar to the “window of implantation”, the time when the endometrium is most able to support trophoblast-endometrial interactions is thought to occur during a short period of time at approximately Days 22–24 of an idealized 28-day cycle [39, 40], and the crosstalk between the blastocyst and the endometrium can only occur during a brief period [41]; a period too late or too early cannot be used. That is, earlier or later transfers did not result in any pregnancies. However, we can be sure that TOP2A affects decidualization.

To elucidate the mechanism underlying TOP2A deficiency-induced decidualization in the endometrium, we subjected the sh-TOP2A and sh-NC T-HESCs, decidualized sh-TOP2A and decidualized sh-NC T-HESCs to mRNA sequencing. Our transcriptome analysis showed that the DEGs were involved in several pathways, including the MAPK signaling pathway, PI3K-Akt signaling pathway, Hippo signaling pathway, and NF-kappa B signaling pathway*.* Several functions of the human endometrium are associated with inflammatory-like responses. During implantation, the expression of various hormones, cytokines, endocrine factors, and growth factors in the endometrium has been reported. Interestingly, NF-κB is a transcription factor involved in the regulation of 400 genes related to implantation, inflammation and immune response [18]. NF-κB is believed to play important roles in endometrial receptivity, first trimester decidua, and the onset of labor in humans [42–44]. Evidence from mammalian models has indicated that NF-κB increases in the pig endometrium between Days 5 and 12 of the estrus cycle before pregnancy [45]. The level and duration of suppression of NF-κB activity in mouse uterine cells appear to be relevant to the timing of implantation [43]. Page M et al. identified increased expression of activating NF-κB components and decreased expression of the inhibitory components in the endometrium at the time of implantation [46]. Our transcriptome analysis showed that the DEGs involved in the NF-κB signaling pathway exhibited notable changes. Our research showed that the protein levels of phospho-NF-κB p65 (p-p65) and phospho-IKK (p-IKK), an activating kinase of NF-κB, increased after treatment with Sh-TOP2A in T-HESCs, indicating that TOP2A affects the NF-κB pathway. In addition, we found that although p-p65/p-IKK was decreased after decidualization, the expression of p-p65/p-IKK in decidualization was increased in the TOP2A knockdown cells compared with the controls, which confirmed that the TOP2A-NF-κB signaling pathway participates in the processes of decidualization and embryo implantation. Moreover, after the application of sh-TOP2A, T-HESCs exhibited greater nuclear accumulation of NF-κB p65, which is critical for binding to the response elements of NF-κB-responsive genes. Thus, altering the TOP2A-NF-κB signaling pathway will be beneficial to decidualization and improve the treatments for patients with RIF.

An insufficiently thickened endometrium indicates a lower likelihood of embryo implantation. The proliferation and decidualization of endometrial stromal cells are very important for embryo implantation. However, the effects of TOP2A, a biomarker for cell proliferation in the reproductive system, especially in the endometrium, remain unclear. In this work, we then generated a TOP2A-deficient mouse model by injection of TOP2A interfering adenovirus through the tail vein on GD2.5 and GD3.5. By applying this approach, we eliminated the effects of TOP2A deficiency before implantation. In the present study, although TOP2A deficiency did not result in implantation failure in the ad-TOP2A mice, the litter size was decreased, and the fetal weights were significantly decreased, which may reflect compromised endometrial receptivity and poor fetal development.

Although ART has improved outcomes for couples struggling with infertility, RIF has emerged as a new challenge. An increasing number of studies have begun to focus on the pathogenesis and treatment of RIF. After several consecutive IVF failures and in agreement with the definition of RIF, patients should undergo three-dimensional ultrasonography, hysteroscopy, or laparoscopy to assess the uterine cavity. Once an abnormality associated with implantation failure is recognized, treatment options including uterine septectomy, removal of intrauterine adhesions, endometrial polypectomy, or myomectomy, particularly the submucous type, and excision of hydrosalpinx should be considered [47–49]. Some women with no clinical manifestations or biochemical abnormalities may have occult endometriosis leading to RIF and lack of effective treatment. Endometriosis is a systemic and reversible inflammatory condition, and treatment of endometriosis has been shown to be beneficial for future fertility and improved pregnancy outcomes[17, 50]. Previous studies have shown that the expression of TOP2A was significantly lower in the endometriosis group than in the control group [34], indicating that the expression pattern of TOP2A in endometriosis is the same as that in RIF in our study. Therefore, we hypothesized that the expression changes of TOP2A may be helpful for evaluating the status and function of the endometrium. Perhaps in future studies, we can explore the expression of TOP2A in the peripheral blood of patients, which may become a convenient, rapid, and effective clinical indicator for evaluating endometrial status and curative effects.

**Fig. 7:**
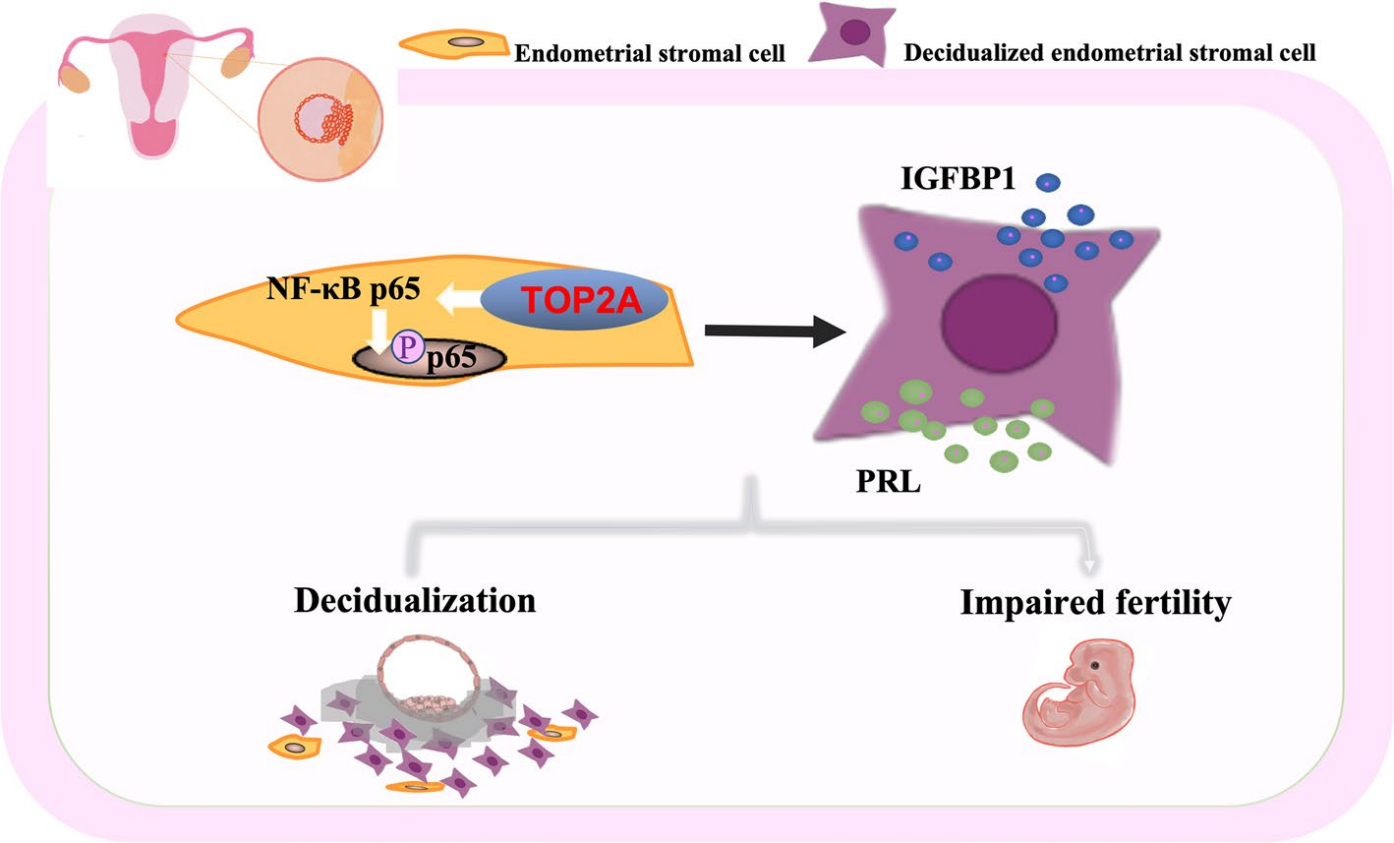
Model for involvement of TOP2A-NF-κB pathway in decidualization. This schematic illustrates our hypothesis that abnormal expression of TOP2A in endometrial stromal cells affect the processes of decidualization and embryo implantation through the NF-κB pathway and result in RIF

To the best of our knowledge, we provide the first evidence that the expression of TOP2A undergoes dynamic changes during the menstrual cycle and that the expression levels of TOP2A are significantly lower in the mid-secretory endometrium of women with RIF. We analyzed possible mechanisms underlying the role of TOP2A in disease pathogenesis. The abnormal expression of TOP2A may affect decidualization and change the “window of implantation”, leading to RIF. We also found that TOP2A may participate in the processes of decidualization and embryo implantation through the NF-κB pathway (Fig. 7). Our results suggest that decreased TOP2A-mediated NF-κB expression plays an important role in women with RIF. We realize that our research is speculative and hope that it will lead to further studies to elucidate the complex mechanisms between the TOP2A/NF-κB signaling pathway and the pathogenesis of RIF.

## Supplementary Information

**Additional file 1: Figure S1.** Bioinformatics analysis of differentially expressed genes in endometrium with recurrent implantation failure from GSE111974. A Volcano plot of all expressed genes in endometrial tissues of the patients with RIF and healthy controls from GSE111974. B The PPI network of the DEGs and the hub gene screening map from GSE111974.

**Additional file 2: Figure S2.** TOP2A inhibition enhances decidualization in T-HESCs. A Western blotting of TOP2A in DMSO-treated group and Etoposide-treated group (1μM) of T-HESCs. B IGFBP1 protein expression in T-HESCs following treatment with DMSO or Etoposide. The cultures either remained untreated or were decidualized for 4 days. C IGFBP1 and PRL mRNA expression in DMSO and Etoposide group. The cultures either remained untreated or were decidualized for 4 days. D CytoPainter phalloidin-iFluor 488 reagent was used to label filamentous actin, and immunofluorescence was used to analyze the morphological transformation of T-HESCs. Decidualized cells were treated with both 8-bromo-cAMP and MPA for 4days. (F-actin, green fluorescence signals; DAPI, blue signals; scale bar = 200μm). One-way ANOVA or Mann-Whitney U test. Data are the mean ± SEM of 3 biological replicates unless stated otherwise. *p < 0.05, **p < 0.01,***p < 0.001. ns, nonsignificant.

## Data Availability

All data generated or analyzed during this study are included in this published article and its supplementary information files. The datasets used and/or analyzed during the current study are available from the corresponding author on reasonable request.
