# Supplementary figures and images for: TOP2A deficit-induced abnormal decidualization leads to recurrent implantation failure via the NF-κB signaling pathway
Source: Reprod Biol Endocrinol. 2022 Sep 22;20:142. doi: 10.1186/s12958-022-01013-1 (PMC9494868; doi:10.1186/s12958-022-01013-1)

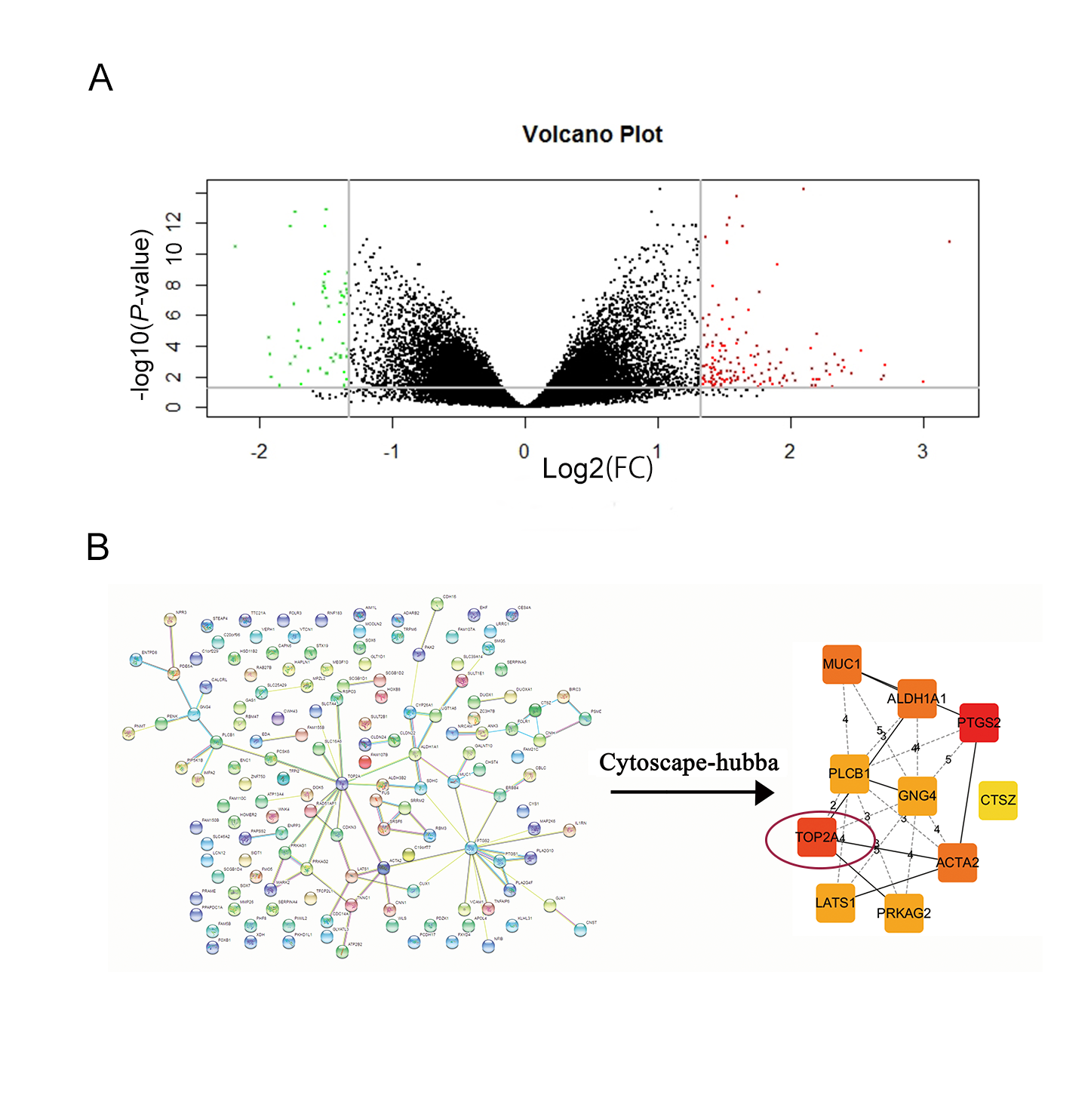

Supplement: Supplementary file 1 — Additional file 1: Figure S1. Bioinformatics analysis of differentially expressed genes in endometrium with recurrent implantation failure from GSE111974. A Volcano plot of all expressed genes in endometrial tissues of the patients with RIF and healthy controls from GSE111974. B The PPI network of the DEGs and the hub gene screening map from GSE111974. [file 12958_2022_1013_MOESM1_ESM.tif]

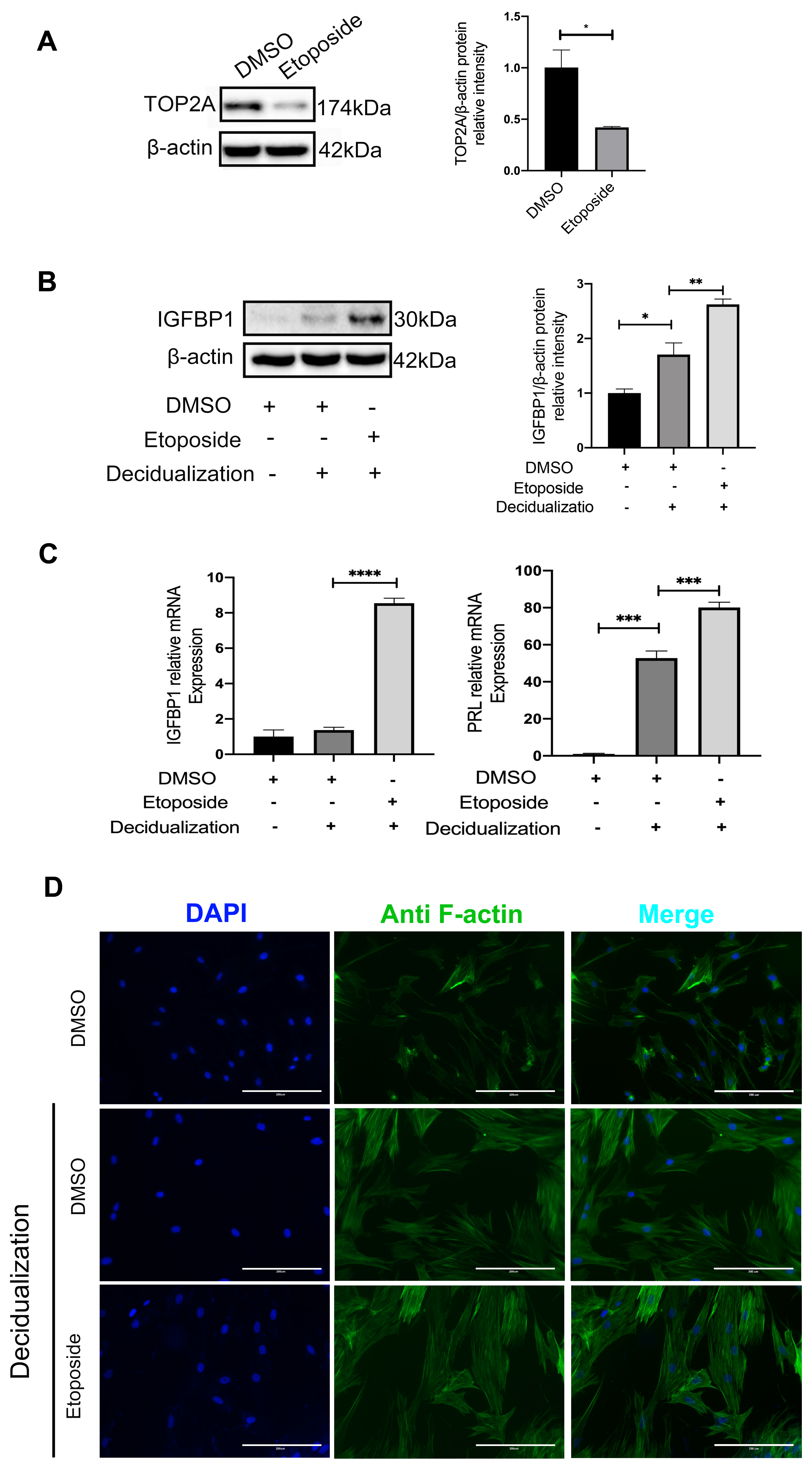

Supplement: Supplementary file 2 — Additional file 2: Figure S2. TOP2A inhibition enhances decidualization in T-HESCs. A Western blotting of TOP2A in DMSO-treated group and Etoposide-treated group (1μM) of T-HESCs. B IGFBP1 protein expression in T-HESCs following treatment with DMSO or Etoposide. The cultures either remained untreated or were decidualized for 4 days. C IGFBP1 and PRL mRNA expression in DMSO and Etoposide group. The cultures either remained untreated or were decidualized for 4 days. D CytoPainter phalloidin-iFluor 488 reagent was used to label filamentous actin, and immunofluorescence was used to analyze the morphological transformation of T-HESCs. Decidualized cells were treated with both 8-bromo-cAMP and MPA for 4days. (F-actin, green fluorescence signals; DAPI, blue signals; scale bar = 200μm). One-way ANOVA or Mann-Whitney U test. Data are the mean ± SEM of 3 biological replicates unless stated otherwise. *p < 0.05, **p < 0.01,***p < 0.001. ns, nonsignificant. [file 12958_2022_1013_MOESM2_ESM.tif]
